# Curcumin and Inflammatory Bowel Disease: Potential and Limits of Innovative Treatments

**DOI:** 10.3390/molecules191221127

**Published:** 2014-12-16

**Authors:** Liza Vecchi Brumatti, Annalisa Marcuzzi, Paola Maura Tricarico, Valentina Zanin, Martina Girardelli, Anna Monica Bianco

**Affiliations:** 1 Institute for Maternal and Child Health—IRCCS “Burlo Garofolo”—via dell’Istria, 65/1, Trieste 34137, Italy; E-Mails: annalisa.marcuzzi@burlo.trieste.it (A.M.); valentina.zanin@burlo.trieste.it (V.Z.); martina.girardelli@burlo.trieste.it (M.G.); annamonicarosaria.bianco@burlo.trieste.it (A.M.B.); 2 Department of Medicine and Surgery and Health Sciences, University of Trieste, Piazzale Europa, 1, Trieste 34137, Italy; E-Mail: paola.tricarico@burlo.trieste.it

**Keywords:** curcumin, inflammatory bowel disease (IBD), inflammation, innovative treatments

## Abstract

Curcumin belongs to the family of natural compounds collectively called curcuminoids and it possesses remarkable beneficial anti-oxidant, anti-inflammatory, anti-cancer, and neuroprotective properties. Moreover it is commonly assumed that curcumin has also been suggested as a remedy for digestive diseases such as inflammatory bowel diseases (IBD), a chronic immune disorder affecting the gastrointestinal tract and that can be divided in two major subgroups: Crohn’s disease (CD) and Ulcerative Colitis (UC), depending mainly on the intestine tract affected by the inflammatory events. The chronic and intermittent nature of IBD imposes, where applicable, long-term treatments conducted in most of the cases combining different types of drugs. In more severe cases and where there has been no good response to the drugs, a surgery therapy is carried out. Currently, IBD-pharmacological treatments are generally not curative and often present serious side effects; for this reason, being known the relationship between nutrition and IBD, it is worthy of interesting the study and the development of new dietary strategy. The curcumin principal mechanism is the suppression of IBD inflammatory compounds (NF-κB) modulating immune response. This review summarizes literature data of curcumin as anti-inflammatory and anti-oxidant in IBD, trying to understand the different effects in CD e UC.

## 1. Introduction

Curcumin, the active yellow pigment of the turmeric spice, is an herb belonging to the ginger family native to India and Southeast Asia. Curcumin is commonly used in Indian traditional cusine and medicine, especially in the treatment of biliary disorders, rheumatism and diabetic ulcers [1].

In more recent years, curcumin has regained interest due to its pharmacological actions and anti-inflammatory, anti-oxidant, anti-tumor, and anti-proliferative properties [1,2,3,4,5,6]. In addition, it is also known for its beneficial effects in neurological diseases by acting as a neuroprotective agent [7]. The principal mechanism, by which curcumin mediates these effects, is connected to the activity of suppression of the nuclear factor kappa-light-chain-enhancer of activated B cells (NF-κB). Furthermore, curcumin activity includes suppression of interleukin-1 (IL-1) and tumor necrosis factor alpha (TNF-α), two main cytokines that play important roles in the regulation of inflammatory responses [6].

For these important activities, curcumin is considered as a valid potential drug treatment in inflammatory bowel disease (IBD). IBD are chronic progressive diseases defined as autoimmune diseases implicated in aberrant and persistent inflammation of the bowel; the two main forms are the Crohn’s disease (CD) and the ulcerative colitis (UC). Although IBD is normally manifested in adulthood, it could onset in childhood even before 2 years of age (early onset child disease, EOCD). This early onset is typically more extensive and characterized by rapid progression, leading to severe repercussion on disease course.

Nowadays there are no therapeutic strategies able to significantly alter the natural history of IBD; instead, the nutritional therapy holds interesting possibilities for the treatment especially considered that conventional pharmacological treatments are discussed for the side-effects and/or adverse events particularly in early onset patients. In this review we summarized literature data of curcumin principal activity, trying to investigate its possible role in the treatment of IBD and to understand the different activities performed in CD and UC.

## 2. Generalities on Inflammatory Bowel Disease

IBD is a multifactorial disorder in which complex interactions among genetic, immune, and environmental factors are involved and represent a group of inflammatory intestinal idiopathic and chronic diseases [8]. The two main forms, CD and UC, overlap as intestinal disease and differ precisely in the clinical, pathogenic and biomolecular features.

UC is a chronic inflammatory disorder restricted to the colon, characterized by abdominal pain, mucosal ulceration, hematochezia and diarrhea. Pediatric patients may present macroscopic skin lesions in the colon, blackwash ileitis and extensive colitis and periappendiceal inflammation [9].

CD is an inflammatory disorder affecting the gastrointestinal tract in both children and adults. All layers of the intestine may be involved and normal healthy bowel can be found between sections of diseased bowel. The inflammation can affect the entire stretch starting from the mouth to the anus and in more complicated cases perianal and strictures fistulas may also arise. Given the variability of the disease localization the possibility to make a rapid and correct diagnosis is not so easy; initially the diagnosis could be done by performing the endoscopic analysis of biopsies from the patient’s gastrointestinal tract; then, a diagnostic workup for staging the disease especially when the onset is very early in pediatric patients is also very important [10]. From the literature it also appears that in a group of pediatric patients with EOCD an upper gastrointestinal and isolated colonic involvement is more frequent and more evident, compared to those patients in whom the disease occurs later [11].

The molecular pathogenesis of IBD is not completely understood, but among contributing factors may be included the bacterial translocation across a defective mucosal barrier and the imbalanced regulation of the intestinal immune response. The patients with IBD are defined considering the parameters of Montreal Classification: the disease onset, the location and the behavior. On the other hand, however, this classification had some limiting criteria for the pediatric patients classification. On this purpose on the classification of Paris has been added, for pediatric classification the growth failure, and the disease onset before 10 years of age (Table 1) [12].

**Table 1:** Pediatric modification of the Montreal classification for inflammatory bowel disease: the Paris classification.

Classification	Montreal	Paris
**Age at Diagnosis**	**A1** <17 year **A2** 17–40 year **A3** above 40 year	**A1a** 0–10 year **A1b** 10–17 year **A2** 17–40 year **A3** > 40 year
**Location**	**L1** terminal ileal ± limited cecal disease **L2** colonic **L3** ileocolonic **L4** only upper disease	**L1** distal 1/3 ileum ± limited cecal disease **L2** colonic **L3** ileocolonic **L4a** upper disease proximal to ligament of Treitz **L4b** upper disease distal to ligament of Treitz and proximal to distal 1/3 ileum
**Behavior**	**B1** non stricturingnon penetrating **B2** stricturing **B3** penetrating **P** perianal disease modifier	**B1** non stricturingnon penetrating **B2** stricturing **B3** penetrating **B2B3** both penetrating and stricturing disease, either at the same or different times **P** perianal disease modifier
**Growth**	not classified	**G**_0_ no evidence **G**_1_ growth delay

### 2.1. Environmental Exposure and Lifestyle

The continuing changes in environmental factors, such as lifestyle, hygiene, medication, diet, affect the IBD onset differently and its course and prevalence is increasing worldwide [13]. The incidence is low in the Pacific region, Asia, South America Africa and Eastern Europe, while is very high in both Northern and Western Europe and in North America. Recent studies, however, have shown an increase of IBD in developing countries and among emigrant populations moving to industrialized societies [14,15]. The interaction between environmental exposure and life styles and IBD is observable, e.g., in the role of hygienic conditions suggesting that less hygienic situations play a protective role and cleaner life conditions are associated with increased rates of IBD, because in rich countries there is a lower rate of enteric pathogens, hot water and sanization resources [16,17]. The role of the breast-feeding leads as well to different conclusions as to whether it plays a protective role or increases the risk by altering the gut microbiota of breast-fed infants [18,19,20,21].

The exposure to antibiotics may influence the risk of IBD development affecting the mechanism that alters the gut microbiome, especially in children [22,23]. In a study of Canadian adults the relationship between the onset of IBD and the amount of antibiotics taken has been confirmed [24]. The lifestyle factors include the living setting and the dietary choices and both may play the role of risk or protective factors on IBD development. In the last decades much attention has also been paid to the living setting, analyzing the effects of the difference between living in an urban setting or in a rural setting on the onset and course of IBD [25]. Exposure to industrial agents and pollution may play an important role increasing the risk of early onset UC and DC [26]. A dietary intake rich in meats and fatty foods increases the risk of CD and a diet based on vegetables, fruits, fish and olive oil was inversely associated with CD. The different aliments can affect the gut permeability and the autoinflammatory response of the mucosa through microbiota alterations [27,28].

All findings evaluated indicate that the relationship between the different environmental factors and lifestyle that can affect CD and UC and the disease onset and course is complicated and the relationship between environment and genetic susceptibility is still unclear.

### 2.2. Genetic Involvement in IBD

The literature increasingly suggests that IBD develops in patients with a certain genetic predisposition and the localization, disease progressions and response to treatments have characteristics that strongly depend on the age of onset [29,30,31,32]. A difficult issue is that the functional relevance of most of the susceptibility genes is unclear and the total of loci identified until now do not account for the total hereditability of IBD. Even given the unproven assumptions that these loci are individually causal and collectively additive, the relative risk conferred by each locus is very small and their overall contribution would account for only 13.6% of CD and 7.5% of UC hereditability [33]. This difficult issue is a problem referred to the missing heritability. In addition to the variants found with the GWAS studies, other genes may be involved and they could be associated with the disease as monogenic pattern of hereditability and this could escape the association analysis [34,35]. There is in addition also the problem of the missing intelligibility, as it is very difficult to define and to make immunological sense of all these loci and pathways, both at the cellular and molecular level. Pediatric IBD onset is increasing; about 20%–25% of IBD patients develop intestinal inflammation during childhood and adolescence and approximately 1% of children below one year develop the disease. Moreover very early onset IBD has an estimated incidence of 4.37/100,000 children and a prevalence of 14/100,000 children [36].

### 2.3. Immune Response and IBD

From different studies can be inferred that an imbalance of pro- and anti-inflammatory factors plays an important role in the pathogenesis of IBD [37,38]. Both disorders, CD and UC, are characterized by immunological responses against (bacterial) antigens but the type of the inflammatory reaction appears to be distinct. Given that in UC there exists neither predominance of interferon gamma (IFN-γ) nor IL-4, while it is clear the up-regulation of IL-5, we can consider it more as a Type 2 Immunity (TH2) disease. On the other hand, as in CD areas of active inflammation with elevated IFN-γ, IL-12 and tumor necrosis factor (TNF) levels are observed, CD is considered a Type 1 Immunity (TH1) disease prototype [39].

It is commonly assumed that T helper cells comprise two subsets, each having different patterns of cytokine production in immune responses: the TH1 and the TH2, both implicated in the regulation of many immune response. The TH1 cells secrete in particular IFNγ, TNFα and TNFβ and IL-2, while the TH2 cells secrete IL-4, IL-5, IL-6, IL-10 and IL-13 [40].

In their study, Pastorelli and colleagues showed an increased expression of IL-33 and IL-1 receptor ST2 in the serum and in the inflamed mucosa of the IBD patients. ST2 exists in two different splice variants leading to the synthesis of ST2L, a transmembrane receptor that confers IL-33’s biologic effects, and sST2, a soluble molecule that likely serves as a decoy receptor for IL-33. From their research it is clear that the system IL-33/ST2 is strongly activated and plays an important role in the pathogenesis of the IBD, also evident in patients with UC. Specifically, it was observed that during active UC, the accumulation of the intraepithelial and intracellular IL-33 as well as the decrease of the ST2L were regulated by TNF. In fact, the anti-TNF treatment of IBD patients, particularly those suffering from UC, modulates the levels of IL-33 and of sST2 [37].

A very recent study also shows that blockage of the IL-33 signal may help in the treatment of UC patients. Specifically this study demonstrates that IL-33 is able to induce the intestinal GATA-3 (master regulatory gene) in the mucosa T cells, thus entrusting to IL-33 a mediating role in the intestinal inflammatory TH2 responses (pathological or not) [41].

By studying how genetic and epigenetic factors influence the age at onset and other clinical features of CD, it is possible to improve the understanding of the disease pathogenesis, in particular through the development of* in vitro* and* ex vivo* models reproducing the interaction between epithelial cells and microbiota. These models are fundamental to study also the development of novel therapeutic approaches aimed to restore a normal balance between immunity and environment. These data contribute to develop disease-specific cellular models based on induced pluripotent stem cells.

Once the disease is started, complete healing is an exceptional outcome, suggesting that some epigenetic changes, such as DNA methylation, may stably affect the way that mucosal immunity respond to intestinal microbiota. Focusing on immune defects as well as excesses in the pathogenesis of IBD may be relevant for therapeutic approaches [42,43].

## 3. IBD and Pharmacological Treatments

The chronic and intermittent nature of inflammation in IBD requires long-term drug treatments in combination or alternation to different drugs. The first aim in treatment is to reduce symptoms and to induce the remission and then to maintain this remission for as long as possible.

The severity, the presence of complications and the goal of the treatment (induction or maintenance of the remission) determine the choice of therapeutic line: aminosalicylates are the first line therapy for mild to moderate IBD; corticosteroids are preferred to use for moderate to severe disease, but are ineffective in the maintenance of remission [44] and then immuno-modulators which generally are not the elective choice due to their slow onset of action and toxicity [45]. Numerous studies have reported on the use of biological factors capable of manipulating the immune and inflammatory responses: inhibitors of T-Cell activation, anti-inflammatory cytokines (IL-10 or IL-11) and inhibitors of pro-inflammatory cytokines including TNF antagonists (infliximab, certolizumab pegol, etanercept, onercept, adalimumab, mitogen-activated protein kinases), inhibitors of PPARs, inhibitors of pro-inflammatory cytokine receptors (anti IL-6 receptor), inhibitors of TH1 polarization (anti-IL-2R antibodies, anti-IL-12, IL-18 and IFNγ) and adhesion molecule inhibitors (natalizumab) [46].

Although there are several potential therapeutic targets considered for the treatment of IBD, currently only a few biologic drugs have achieved any significant results and are approved for treatment, such as infliximab that has been introduced into clinical routine in the United States in 1988 and continues to be effective.

### Adverse Effects of IBD Treatments

The aminosalicylates are effective in controlling the inflammation, but may have adverse effects such as nausea, vomiting, heartburn, diarrhea and headache. The side effects of the corticosteroids are weight gain, acne, facial hair, hypertension, diabetes, bone mass loss and increased risk of infections.

The use of immunosuppressant drugs (thiopurines, methotrexate, tacrolimus, thalidomide, cyclosporine and infliximab) is effective in the treatment of IBD (active or quiescent CD or in cases of steroid dependent UC), as well in pediatric patients [47,48], but these drugs are not without adverse effects, sometimes also serious as the case of methotrexate, which may cause dyspepsia, alopecia, myelosuppression, abdominal pain, headache and arthralgia [49]. Thalidomide, that acts as an inhibitor of TNFα synthesis, besides being a teratogen, could lead to peripheral neuropathy, dizziness and allergic reactions [47,50]. The use of tacrolimus has shown an elevated risk to develop adverse effects in UC patients, including the most common, finger tremors. The anti-TNFα molecules, including infliximab and adalimumab, cause reactivation of latent infections, cutaneous reactions (skin eruptions and macules), systemic and hematological complications, allergic events and local side effects [51,52]. Recently Lakatos* et al.*, suggested that the use of biological drugs for a long time may increase the risk to develop malignancies such as the non-Hodgkins’s lymphoma [53].

It has also been seen that therapy with biological agents (anti-TNFα), as well as the use of immunosuppressants and/or the repeated use of corticosteroids, make patients much more susceptible to endemic and opportunistic infections by bacteria, fungi, parasites or pathogens viruses [54].

Cyclosporine acts by inhibiting the production of IL-2 by activated T lymphocytes and it is used in the case of severe UC, but it is related to manifestations such as hypertension, impaired renal function and neurotoxicity (tremor or paresthesia), as well as minor adverse effect such as fever, headaches and diabetes mellitus [55].

As regards children and adolescents, researchers must bear in mind that the disease involves the physical but also the psychological level and drug treatments can affect the quality of life. Even if the strategies that involve the steroids use in children are preferable, one study performed in children suffering from Crohn’s disease has shown that a short course of polymeric diet was more effective then corticosteroid treatments [56]. Conventional medications used in the treatment of symptoms of IBD consist of anti-inflammatory and immuno-modulator drugs as summarized in Table 2 [44,45,46,47,48,49,50,51,52,53,54].

## 4. Curcumin: Potential and Limits

Curcumin (diferuloymethane) is the most active component of the plant *Curcuma longa*, belonging to the family Zingiberaceae also known as turmeric, commonly employed as a natural food additive. It is an indigenous plant of India, but is also cultivated in other countries such as China and Sri Lanka [57]. Used in Indian and Chinese traditional medicine, it has been described as an anti-inflammatory, antioxidant, pro-apoptotic, chemopreventive, antitumor and antimicrobial compound, as reported in Table 3 [58,59,60,61,62,63,64,65,66,67,68,69,70,71,72,73,74,75,76,77,78,79,80,81,82,83,84,85,86,87,88,89,90,91,92,93,94,95,96,97,98,99,100,101,102,103,104,105,106,107,108,109,110,111,112,113,114,115,116,117,118,119,120,121,122,123,124,125,126,127,128,129,130,131,132,133,134,135,136,137,138,139,140].

Several studies have described the strongly link within these beneficial properties; indeed they don’t show different and separated effects, but they are related each other as a consequence of some specific characteristic. As an example, the anti-oxidant effect of curcumin and analogues was related to their anti-tumor and anti-inflammatory mechanism, as already described in recent studies [141,142,143].

Curcumin has been proposed to be a therapeutic molecule in various illnesses such as arthritis, cancer, diabetes, cardiovascular diseases, liver fibrosis, gall stone formation, neurological disease and inflammatory bowel disease [144,145]. The clinical development as a therapeutic drug is limited due to its poor aqueous solubility, poor absorption, biodistribution, rapid metabolism and fast elimination [146,147]. In the last years various natural and synthetic analogues of curcumin have been synthesized to improve bioavailability problems increasing its therapeutic potential, but their effectiveness is still controversial [148].

In spite of all this, the oral administration of the drug allows for an active level of curcumin in the gastrointestinal tract, making it a good candidate for the treatment of the diseases in this anatomical site [149,150,151].

How curcumin exerts its pleiotropic effects has been thoroughly investigated and a vast array of targets has been assumed to play a role in the disease pathogenesis. Some molecular targets include transcription factors, inflammatory cytokines, enzymes and the epigenetic modulation which modulate histone deacetylases, histone acetyltransferases, DNA methyltransferase I and miRNAs [80].

**Table 2:** Characteristics of inflammatory bowel disease.

	Localization	Symptoms	Cytokine Inflammation	TH1/TH2	Treatments
**Crohn’s Disease (CD)**	Deep layers of the intestinal wall, the ileum, the first part of the colon, esophagus, stomach and duodenum	Pain in the abdomen, diarrhoea, weight loss, rectal bleeding and fever	Interferon gamma (IFN-Y), Interlukin 12 (IL-12), Tumor Necrosis Factor (TNF)	TH1 disease	Anti-inflammation drugs, corticosteroids, immunomodulators and biologic treatments
**Ulcerative Colitis (UC)**	Inner lining of the colon (large interstine) and rectum	Diarrhoea, abdominal cramps, rectal bleeding, frequent fever and nausea	Interlukin 5 (IL-5), Interlukin 33/Interlukin 1, Receptor ST2 (IL-33/ST2)	TH2 disease	Aminosalicylates, corticosteroids, immunomodulators and biological treatments

**Table 3:** Molecular targets of curcumin and relative effects and diseases involved.

Targets of Curcumin	Effects of Curcumin	Diseases Involved
Activation of redox-regulated transcription factor Nrf2 that induces heme oxygenase 1 (HO1) paraoxonase 1 (PON1) and GSH 3 [58,60,61,62]	Free-radical-scavenging activity	Chronic inflammatory diseases
Inhibition of DNA-binding of STAT3 3 [63]	Anti-inflammatory activity	Chronic inflammatory diseases
Reduced phosphorylation of cytosolic phospholipase A2 (cPLA2) limiting the arachidonic acid availability [64]	Anti-inflammatory activity	Chronic inflammatory diseases
Reduced phosphorylation of IκB [65]	Anti-inflammatory activity	Chronic inflammatory diseases Cancer
Inhibition of the transcription factor Nf-κB [65,66,67,68,69,70,71,72]	Anti-inflammatory activityAnti-oxidant activity Tumor suppressive activity	Chronic inflammatory diseases Cancer
Inhibition of mRNA levels of COX2 and iNOS [73,74,75,76]	Anti-inflammatory activity Tumor suppressive activity	Chronic inflammatory diseases Cancer
Inhibition of matrix metalloproteinases MMP-9 and MMP-2 [72,77,78, 79]	Tumor suppressive activity (Anti-inflammatory activity)	Cancer (Chronic inflammatory diseases)
Inhibition of histone deacetylases (HDACs) and acetyltransferases (HATs) activity [80,81,82,83,84,85,86,87,88]	Gene regulation	Cancer
Up-regulation and down-regulation of micro RNA (22, 199, 186, 203) [80,89,90,91]	Pro-apoptotic activity Tumor suppressive activity	Cancer
Activation of caspase 3, 7, 8 and caspase 9 [92,93,94]	Pro-apoptotic activity	Cancer
Increased cleavage of poly (ADP-ribose) polymerase (PARP) [67,95]	Pro-apoptotic activity	Cancer
Up-regulation of several tumor suppressor genes [95,96,97,98,99]	Tumor suppressive activity	Cancer
Up-regulation of different proapoptotic genes [100,101,102,103]	Pro-apoptotic activity Tumor suppressive activity	Cancer
Inactivation of several oncogenes [104,105,106,107,108,109,110,111,112]	Tumor suppressive activity	Cancer
Down-regulation of different antiapoptotic genes [113,114]	Pro-apoptotic activity Tumor suppressive activity	Cancer
Inhibition of angiogenesis suppressing VEGF, Akt and PI3K [65,115,116,117,118]	Tumor suppressive activity	Cancer
Inhibition of enzymes of phase I reactions [119,120,121]	Tumor suppressive activity	Cancer
Activation of enzymes of phase II reactions [122,123,124,125,126,127,128]	Tumor suppressive activity	Cancer
Down-regulation of androgen receptor (AR) [110,129,130,131]	Tumor suppressive activity	Cancer
Repressed N-methyl-D-aspartate (NMDA) receptor-mediated Ca^2+^ [132,133,134,135,136]	Protection from excitotoxicity	Neurodegenerative diseases
Reduced oxidative mitochondrial damage [137,138,139,140]	Antioxidative activity	Neurodegenerative diseases

**Figure 1:**
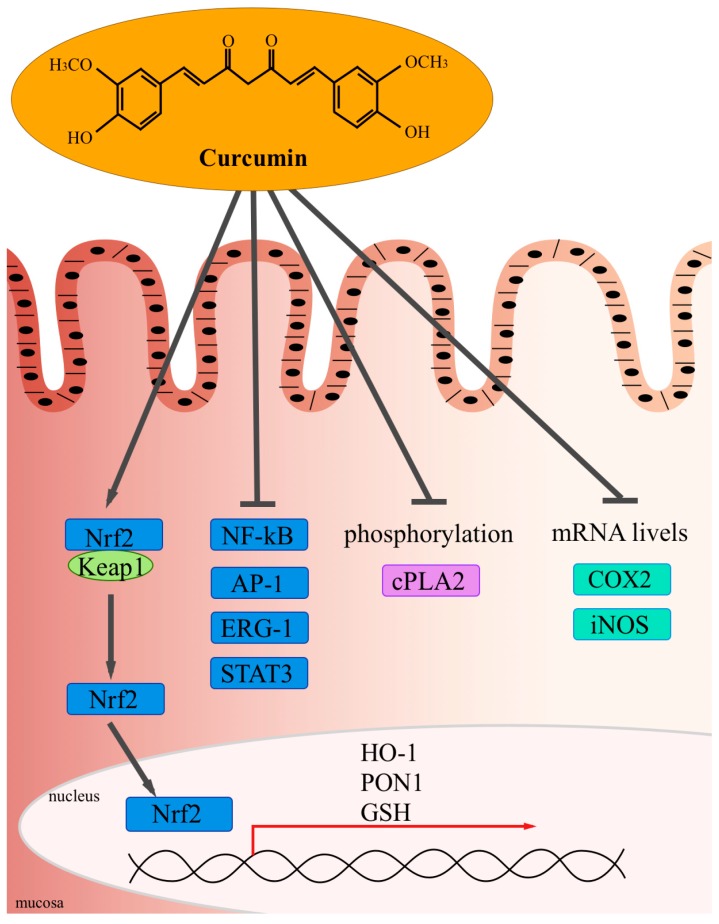
The curcumin activity in mucosal.

In idiopathic inflammatory bowel diseases the persistent inflammation depends partially on the activation of NF-κB signaling cascade level or other molecular targets, as indicated in Table 3, and part of the pleiotropic effect of curcumin seems to due to inhibition of this pathway. *In vivo* and* in vitro* studies exhibit a different reaction to curcumin in both IBD form, CD and UC, probably depending on the kind of immune dysregulation involved, as shown in Figure 1 [152,153,154,155].

### Experimental Studies and Clinical Trials

In their study Billerey-Larmonier* et al*., showed the effect of dietary curcumin in two mice strains (BALB/c and SJL/J) with chemically induced colitis. An improvement in the pathological condition of BALB/c mice, exhibiting a mixed TH1/TH2 response, was detected, while a lack of benefits was revealed in SJL/J mice characterized by TH1 response, suggesting a role of curcumin in regulating the immune response [152].

Other studies showed a weakened colonic inflammation in induced colitis mice and rats due to inhibition of NF-κB pathway, p38 mitogen-activated protein kinases (MAPK) activity and reduction of pro-inflammatory TH1 cytokine response, resulting in suppression of inducible nitric oxide synthase and lower neutrophil recruitment [156,157]. Furthermore, in mice with spontaneous development of intestinal inflammation the administration of the Indian spice showed a reduced inflammation in histological colonic pattern [158]. In the human colonic mucosa of IBD patients a reduced MAPK activity was detected, while IL-10 was increased and IL-1β reduced [154]. Although in mice experiments curcumin was administered intraparenterally, the oral administration in human patients highlighted improvements on the overall IBD situation, detecting however, a similar response to curcumin as adjuvant IBD therapy in patients with CD and UC [159,160]. The co-administration of curcumin with conventional drugs was also shown to be safe and well-tolerated in IBD pediatric patients, and in fact no clinically significant side effects were reported [161].

Keeping in mind that CD is associated with a TH1/TH17 cell mediated response, while UC is associated with atypical TH2 response, clinical trials, unlike* in vivo* studies, suggest that curcumin could act on a common pathway shared by the two immune responses, probably NF-κB.

To evaluate the curcumin efficacy in IBD disease a study on 10 IBD patients was conducted: five patients suffered from CD and five were affected by Ulcerative Proctitis, a mild form of UC. For this form of UC patients were administered for the first month of therapy 550 mg of curcumin twice daily and for the second month the same dose, but three times a day. After therapy a significant reduction of both the symptoms and the inflammatory indices was evident. To CD patients instead 360 mg were administered three times per day for the first month and four times daily for the second and third month. Only four patients completed the study and in them an evident reduction of both the CD activity index and some indicative parameters was observed [162].

Another study was carried out in an adult woman (60 years) suffering from UC and enteropathic arthropathy. After having tried all medications including all possible combinations and after refusing treatments with biological agents because of possible side effects, she agreed to also take 500 mg of curcumin daily with 40 mg of prendnisone. After a year of treatment, improvements were evident to the extent that there were no marked ulcerations and biopsies showed a chronic inactive UC [163].

Considering the positive effects of curcumin obtained in these studies described above and others performed in patients suffering from IBD, curcumin could be an alternative and/or an additional treatment in controlling both CD and UC disease [151]. We report in Table 4 the main clinical trials, ongoing and concluded, evaluating the efficacy of curcumin in IBD.

## 5. Curcumin and Inflammation

The NF-κB transcription factor family plays a key role in several cellular functions (inflammation, apoptosis, cell survival, proliferation, angiogenesis and innate and acquired immunity) as well as in regulating the expression of more than 500 different genes involved in inflammatory and immune responses [155,164]. Many molecules are involved in the inflammatory response regulated by NF-κB pathway (e.g., TNF, IL-1, IL-6, IL-8, IL-10) as well as tissue destructive enzymes (e.g., matrix metalloproteinases and prostaglandins). TNF is the most promptly released cytokine upon injury and, through the interaction with its receptors, it regulates the production of pro-inflammatory and anti-inflammatory cytokines [165]. Therapies that target rate-limiting steps like TNF (such as infliximab), have markedly improved the autoimmune disease progression in IBD. Moreover NF-κB plays a pivotal role in the regulation of the adaptive phase and in the resolution of the inflammation through apoptosis mechanisms, including the pyroptosis IL-1β dependent one [166,167].

All these findings considered, the activity of curcumin in this context is very important, because it mediates its effects through modulation NF-κB and pro-inflammatory cytokines, such as IL-1β, TNF-α and IL-6 [168,169]. The transcriptional factors NF-κB as well as growth factor and growth factor receptors, protein kinases, adhesion molecules and enzymes are molecular targets for curcumin activity [170].

**Table 4:** Clinical trials to assess the efficacy of curcumin in IBD.

ClinicalTrials.gov Identifier	Number of Patients (Age)	Disease	Doses of Curcumin	Phase
NCT01320436	50 (18 to 70 years)	Ulcerative colitis (Disease activity score of >5 and ≤13 according to the Simple clinical colitis activity index (SCCAI)	Patients allocated for this arm will receive 5ASA medication (as advised by their treating physician) + 3 capsules (820 mg each) curcumin twice daily after meals.	3
NCT00889161	11 (8 to 18 years)	Inflammatory bowel disease (mild disease or in clinical remission) [160]	Initial dosage of 500 mg twice a day for 3 weeks. Using the forced dose titration design, dose will be titrated up to 1 g twice a day at week 3 for a total of three weeks and then titrated again to 2 g twice a day at week 6 for three weeks	1
NCT00793130	30 (18 to 75 years)	Mild or moderate Ulcerative Colitis	Dietary Supplement: Coltect Two tablets twice daily (BID) during the 2 months of the study. Each tablet contains 500 mg Curcumin, 250 mg Green tea and 100 μg Selenomethionine.	Unknown
NCT01647412	40 (10 to 17 years)	Crohn’s Disease (Moderate to severely active CD, as defined by a PCDAI score >30 and	The experimental group will receive the exclusion diet and nutraceutical therapy (DNT) and daily subcutaneously administered recombinant human growth hormone (rhGH) for the first 26 weeks. After 26 weeks this group will continue on the exclusion diet nutraceutical therapy for the remaining 26 weeks of the study.	2
	89 (13 to 65 years)	Ulcerative Colitis (patients in remission of disease) [148]	Oral curcumin (2 g/day; 1 g morning and evening, after meals)	Concluded

## 6. Curcumin Analogues and Nanoformulations

This natural bioactive component has shown a wide range of biological properties and pharmacological actions, suggesting interesting clinical applications, but researchers must face the other side of the coin, that is all those factors contributing to the low bioavailability, the poor solubility (*i.e*., 0.4 mg/mL at pH 7.3) and absorption, and the rapid metabolic elimination by reduction and conjugation [171] causing limitations to the clinical applications. In order to solve this problem, there is a need to develop curcumin analogues and nanoformulations with higher metabolic stability than the original compound [172,173,174].

### 6.1. Curcumin Analogues

Recently several studies, as those experimental *in vivo* evaluating the tumor cell viability, have demonstrated that compounds analogous to curcumin have the same beneficial properties of the original compound and, at the same concentration, even a better effect [175].

Curcumin analogues, such as dimethoxycurcumin or novel water-soluble curcumin derivatives have shown good pharmacological effects in metabolic disorders and in diabetes mellitus [176,177]. The major biochemical characteristics needed in analogues are stability, good pharmacokinetic properties, drug release in the correct site and decreased fluctuations [178]. Moreover recent studies support previous evidence that the biological activity of analogues of curcumin (as an example, 2,5-bis(4-hydroxy-3-methoxybenzylidene)cyclopentanone) was better than that of curcumin: the antioxidant and anti-cyclooxygenase activities of this compound are 2- and 7-times higher, and the anti-inflammatory activity 5-times higher than those of curcumin at a dose of 20 mg/kg, p.o. This compound, indeed, potently inhibits histamine release by altering some intracellular signaling events in mast cells and will be a good candidate for an anti-allergic and anti-inflammatory drug [179,180,181,182].

### 6.2. Nanoformulations

Furthermore, to overcome the low aqueous solubility of curcumin as a therapeutic agent, many technologies have been developed and applied. In particular several studies have described the positive results obtained from the design and the development of nano-sized delivery systems for curcumin, including liposomes, polymeric nanoparticles and micelles, conjugates, peptide carriers, cyclodextrins, solid dispersions, lipid nanoparticles and emulsions [183,184].

Literature data report preliminary promising results obtained by experimental *in vitro* and* in vivo* studies, through the development of specific curcumin delivery systems protecting against the fast degradation and targeting the inflamed colon. The compound was encapsulated in polymeric pH-sensitive nanoparticles to obtain a selective and specific delivery to the inflamed mucosa. Nano-sized drug delivery systems represent an efficacious strategy against the inflammatory system in IBD treatment [185,186]. In particular recent studies show the effective anti-inflammatory properties (myeloperoxidase activity, a measure of neutrophil infiltration, and TNFα secretion),* in vitro* and* in vivo*, obtained by curcuma encapsulated in polymeric pH-sensitive nanoparticles for a selective and specific delivery of curcumin to the inflamed mucosa [186,187].

The nanoformulation that seems to be the better solution as methods to deliver the curcumin in IBD condition is cyclodextrin-curcumin complex: the results obtained* in vitro* and* in vivo* confirmed that hydroxypropyl-β-cyclodextrin-curcumin complex represents a valuable innovative therapeutic approach for IBD treatment [187]. Although the nanoformulations have shown a good level of safety it is necessary to pay attention to their potential toxicity, especially with repeated administrations at high dosage [176].

## 7. Conclusions

Curcumin is a natural compound that reduces the development of chronic experimental colitis and alleviates the inflammatory response whose precise modes of action is still unclear, and it seems likely that its molecular targets differ according to cell and disease system. Several studies have demonstrated the promising role of curcumin as a novel therapy for children and adults with IBD.

To date a precise understanding of the effective dose, safe regimental therapy, and mechanism of action for the use of curcumin in the treatment of IBD is unknown, but there is abundant evidence proving its effects on the NF-κB pathway and p38 MAPK in the intestinal mucosa.

The key role played by curcumin in the diet and its implications for the quality of life of IBD patients should be studied because preliminary data obtained in clinical trials are very encouraging. The pleiotropic role of curcumin in IBD pathogenesis and range severity of phenotype is very remarkable.

We conclude that large-scale, double-blind trials need to be conducted to establish the role of curcumin in the treatment of IBD. The parameters crucial to be included in the study are disease onset, age of patients, pharmacological assumptions and diet interaction, administration with respect to the inflammation phase (acute or in regression). In case of nanoformulations, clinical trials are also required to establish not only the efficacy, but also the safety in case of repeated use. In conclusion we think that it is necessary to deepen if, how and how much curcumin is useful for preventing the recurrence of IBD by modifying the patient’s diet in remission periods and/or for decreasing the mucosal inflammation in the acute phase.
